# A sex-linked supergene with large effects on sperm traits has little impact on reproductive traits in female zebra finches

**DOI:** 10.1098/rspb.2023.2796

**Published:** 2024-03-27

**Authors:** Katherine Assersohn, Oscar Morton, Jon Slate, Nicola Hemmings

**Affiliations:** ^1^ School of Biosciences, University of Sheffield, Sheffield S10 2TN, UK; ^2^ Department of Plant Sciences and Conservation Research Institute, University of Cambridge, CB2 3EA, UK

**Keywords:** reproduction, sex chromosomes, heterozygote advantage, inversion polymorphism, *Taeniopygia guttata*

## Abstract

Despite constituting an essential component of fitness, reproductive success can vary remarkably between individuals and the causes of such variation are not well understood across taxa. In the zebra finch—a model songbird, almost all the variation in sperm morphology and swimming speed is maintained by a large polymorphic inversion (commonly known as a supergene) on the Z chromosome. The relationship between this polymorphism and reproductive success is not fully understood, particularly for females. Here, we explore the effects of female haplotype, and the combination of male and female genotype, on several primary reproductive traits in a captive population of zebra finches. Despite the inversion polymorphism's known effects on sperm traits, we find no evidence that inversion haplotype influences egg production by females or survival of embryos through to hatching. However, our findings do reinforce existing evidence that the inversion polymorphism is maintained by a heterozygote advantage for male fitness. This work provides an important step in understanding the causes of variation in reproductive success in this model species.

## 1. Introduction

Reproductive success is a central determinant of evolutionary fitness, so we expect traits important for reproduction to be under strong directional selection. Despite this, reproductive output often varies considerably between individuals, and the genetic basis of this variation is not well understood [1–3]. Chromosomal inversions are widespread across taxa, and inversion polymorphisms are increasingly recognized as important in the maintenance of genetic variation [4,5]. Estrildidae finches like the zebra finch (*Taeniopygia guttata*) are particularly prone to inversions [6,7], making them useful for studying the relationship between inversions and the maintenance of reproductive trait variation.

**Figure 1.**
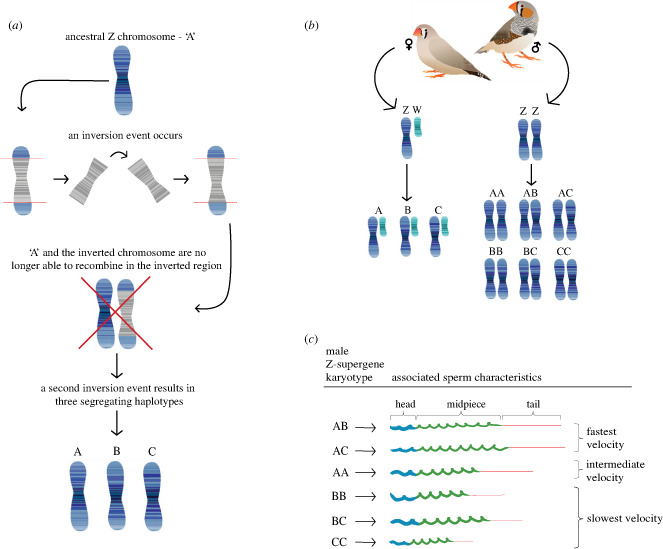
(*a*) Schematic illustrating the formation of the zebra finch Z chromosome inversion polymorphism. A large inversion (i.e. the complete end-to-end reversal of gene order) occurred on the ancestral Z chromosome ‘A’, generating an alternate Z chromosome haplotype. The ancestral and inverted haplotype were then unable to recombine in the inverted region (commonly referred to as a ‘supergene’), resulting in a build-up of genetic differences over time. A second inversion independently generated a third haplotype. The grey region illustrates the inverted non-recombining region. Note that we currently do not know the order in which B and C were generated, whether all haplotypes were generated from A or sequentially from one another, or whether additional inversions were involved. Chromosome illustrations do not represent accurate maps. (*b*) Schematic illustrating all inversion genotypes carried by females and males. Female birds are heterogametic, and so female zebra finches carry a single Z haplotype (inherited from their father). Males are homogametic and carry one of six possible karyotypes. (*c*) Schematic illustrating the effect of Z karyotype for male sperm characteristics (scale is approximate). Differences between Z haplotypes almost entirely explain the variation in sperm morphology and swimming speed in the zebra finch [8]. All three haplotypes are maintained at stable frequencies in the population, probably because heterokaryotypic males carrying one A haplotype and one alternative haplotype (i.e. AB or AC males) have the fastest and most successful sperm, which are associated with longer midpieces and relatively long overall length. AA males have intermediate velocity associated with long tails but short midpieces, whereas BB, BC and CC males have sperm characterized by relatively short tails, short overall length, and the slowest velocity. Sperm speed categories are based on velocity data from Kim *et al*. [8].

Like all birds, zebra finches have a ZZ/ZW sex chromosome system whereby females are the heterogametic sex (ZW). The zebra finch Z chromosome houses a polymorphic inversion consisting of (at least) three segregating haplotypes: A, B and C (figure 1*a,b*) [8,9]. Recombination is highly suppressed within the inverted region in heterozygous males, so mutations that arise on a given genetic background (i.e. haplotype) cannot recombine onto other haplotypes, but instead are preserved and inherited together as a single unit [10,11]. When inversions link multiple loci that act jointly to encode complex phenotypes in a balanced polymorphism, they are commonly referred to as a ‘supergene’ [8,10,12].

The zebra finch Z inversion polymorphism is large, containing over 600 genes and spanning 86% of the chromosome [6,13]. The inversion's molecular evolution is yet to be fully determined, and while A is likely to be the ancestral haplotype, it is unclear the order in which B and C arose, and whether they were both generated from A or sequentially from one another. Each haplotype has accumulated large genetic differences over time, and all three are maintained at stable frequencies in both captive and wild populations (frequencies of A, B and C respectively: 0.378, 0.408 and 0.215 in the captive population studied here; and 0.730, 0.199 and 0.122 in the wild) [8]. Importantly, the male inversion karyotype is known to be responsible for almost all the variation in sperm morphology and swimming speed in this species (figure 1*c*), with important consequences for male fertilization success under sperm competition [8]. Sperm morphology is extremely variable between inversion karyotypes, and heterokaryotypic males (AB or AC karyotypes) have the fastest and most successful sperm under a competitive scenario (figure 1*c*). This suggests that all three haplotypes, including those associated with relatively poorly performing sperm (e.g. BB, BC and CC males), are being maintained in the population at least in part by a heterozygote advantage for male reproductive success (figure 1*c*) [8].

Whether the observed overdominance for male reproductive success is the only force maintaining all three inversion haplotypes in this system remains unknown. Females may also influence the maintenance of the inversion polymorphism, some (non-exhaustive and non-mutually exclusive) examples include sexually antagonistic selection, variation in male-female compatibility, or through paternity bias based on male karyotype [4,10]. Females could bias paternity either before copulation via assortative mating; or post-copulation, for example via selective sperm storage, reduced egg investment, reduced incubation effort, or by modifying brood sex ratio [14–19]. Female zebra finches have been known to modify egg investment in response to partner quality [20,21], and can facultatively adjust offspring sex ratio depending on female condition [22]. In some other passerines, females have also been shown to adjust clutch sex ratio depending on mate quality [16,23]. These mechanisms of paternity bias could potentially act to maintain the inversion polymorphism by reinforcing the existing heterozygote advantage, either through propagating the benefits associated with certain male karyotypes, or by offsetting the disadvantages associated with other karyotypes, or both. However, while previous work has found evidence for strong additive effects of Z-chromosome inversion genotype on several morphological traits [6], whether females can detect and respond to these signals of male inversion karyotype is unknown.

The direct effects of the inversion polymorphism on female fitness have also not yet been fully explored. Sexually antagonistic loci–loci that contain alleles which benefit one sex at the detriment of the other, are predicted to be particularly prevalent in sex-linked regions, owing to how the unique pattern(s) of sex chromosome inheritance affect the evolutionary dynamics of sexually antagonistic selection [24–26]. Whether a Z-linked sexually antagonistic allele will reach fixation is dependent on both the sex in which it benefits, and its dominance coefficient [24–29]. For example, if recessive sexually antagonistic alleles have accumulated within the Z chromosome inversion, they could potentially act to maintain the polymorphism because haplotypes with alleles that are detrimental to males but beneficial in females can reach high frequencies (recessive alleles are always expressed in females). Conversely, the higher dose of the Z in males, coupled with the fact that dosage compensation (the equalization of gene expression between the autosomes and the sex chromosomes in the heterogametic sex) is incomplete on the avian Z, suggests that fully or partially dominant Z-linked male-beneficial alleles could be positively selected, even if sexually antagonistic [24,25,27,30–33]. To determine whether sexually antagonistic selection could be contributing to the maintenance of the Z-chromosome inversion polymorphism, it is necessary to determine whether female reproductive traits also vary based on inversion haplotype, as well as measure the survival probability of offspring with different combinations of haplotypes.

Here, we use an extensive long-term breeding database with data from 1716 genotyped captive zebra finch individuals to analyse the effects of both male and female inversion haplotype (individually and in combination) on reproductive traits including egg production (a female-specific reproductive trait) and survival rates of embryos through to hatching. This work provides an important step in improving our understanding of the consequences of inversion polymorphisms for reproduction, and more broadly the causes of variation in reproductive success in birds from both the male and female perspective.

## 2. Methods

### (a) Animals

All zebra finches belonged to a domesticated population maintained at The University of Sheffield between 1985 and 2016. Birds from this population were separated physically (but not visually or acoustically) by sex unless breeding, in which case a single pair was housed together without access to other individuals and with no opportunity to engage in extra pair copulations. Pairs were selected by researchers and no natural mate choice was permitted. Previously mated females were rested for at least two weeks before re-pairing with a new male to ensure sperm from the previous male was fully depleted from her sperm storage organs (the maximum duration of sperm storage is 13 days in the zebra finch [34]). Paternity was therefore known conclusively for every egg, and sperm did not have to compete for fertilization of the ova. All deceased birds were preserved in −20°C freezers and accessed later for tissue sampling and DNA extraction. All sampled birds were adults.

### (b) DNA extraction

Z chromosome inversion karyotype was already known for 197 male birds from a previous study [8]. Tissue samples (brain or toe tissue) were dissected from an additional 1785 frozen specimens (1071 females and 714 males). Tissue samples were stored at room temperature in 100% ethanol until extraction. DNA was extracted using a standard (plated) ammonium acetate method [35], and DNA samples were stored in a low TE buffer (Tris-HCL (1 M) and EDTA (0.5 M)) at −4°C and later quantified using a FLUOStar fluorometer before diluting to a concentration of 5 ng µl^−1^.

### (c) Single nucleotide polymorphism typing

Samples were typed for their single nucleotide polymorphism (SNP) genotypes using kompetitive allele specific polymerase chain reaction (KASP)-genotyping chemistry on an LGC SNPLine system [36] in The University of Sheffield's Molecular Ecology Laboratory. A total of nine SNPs were chosen from a zebra finch high density 600k SNP chip. These SNPs demonstrated the highest fixed allelic differences between haplotypes (see Kim *et al*. [8] for details). Individual assays for each SNP were designed using LGC Genomics Ltd guidelines. Genotype calling was performed using cluster analysis in the software Kraken [37]. Diagnostic SNP data obtained from Kim *et al*. [8] was used to call SNP genotypes for each individual [38], and genotype calling was successful for 98% of samples. Unsuccessful calling for 2% of samples was most often owing to amplification failure, indicating insufficient DNA present in the sample. A total of 1753 individuals were typed (1950 including the additional males from the Kim *et al*., dataset). Owing to some uncertainty in the breeding record (missing or ambiguous data), 12% of individuals (233) were removed, leaving a total of 1716 individuals, including 792 males (AA: 139; AB: 229; AC: 111; BB: 168; BC: 115; CC: 30), 924 females (A: 340; B: 376; C: 208) and 1319 unique pairings in the final datasets (see the electronic supplementary material, table S2 for sample sizes of parental genotype combinations) [38].

### (d) Measures of reproductive success

We investigated the effect of female inversion haplotype, male inversion karyotype and the combination of male and female genotype on five reproductive traits:

(i) egg production: measured as the number of eggs laid per clutch (all eggs laid including infertile eggs) (n clutches: 3041; n pairs: 1319; n eggs: 12292);
(ii) egg fertility and early embryo development: measured as the proportion of eggs (per pair) that were fertilized and survived past 3 days of incubation, relative to eggs that were either unfertilized or died within the first 3 days of development (*n* pairs: 454). Failed fertilization and death during early development were not distinguished from one another because, prior to 2008 (when microscopic techniques were developed to accurately determine the fertility status of undeveloped eggs [39]), egg fertility was primarily determined by candling (i.e. shining a light through the eggshell to visualise the contents without opening the egg). Zebra finch embryonic development is only reliably visible via candling after 3 days of incubation, therefore eggs with no visible sign of development by 3 days could have either been unfertilized, or fertilized but the embryo died very early. Eggs with visible signs of development by day 3 of incubation could be definitively classified as fertilized and had survived the very early stages of development (prior to the formation of blood vessels). Both fertilization failure and early-stage embryo mortality are more likely to be linked to genetic problems (e.g. sperm-egg incompatibility, aneuploidy), parental age effects or variation in female receptivity to sperm, compared to later stage mortality [40];
(iii) hatching success of developing eggs: measured as the proportion of eggs (per clutch) that showed obvious signs of development on candling (i.e. were fertilized and developed to at least day 3 of incubation) and went on to successfully hatch, relative to those in which the embryos died at a relatively late stage of development (i.e. after day 3 of incubation), resulting in hatching failure (*n* pairs: 460; *n* clutches: 1528). Compared to early-stage embryo mortality, late-stage mortality is more likely owing to environmental conditions in the nest and/or inadequate parental care during incubation [40]. Any effect of inversion haplotype or parental haplotype combination on late-stage embryo development is therefore likely to occur through selective parental investment, rather than direct genetic effects;
(iv) offspring sex ratio: measured as the deviation of offspring sex ratio from a 50 : 50 expectation (*n* clutches: 1769, *n* offspring: 880). Only offspring that survived to 100 days were included in the sex ratio data as sex was assigned based on plumage traits at sexual maturity; and
(v) offspring genotype: measured as the consistency of offspring genotypes with Mendelian expectations. For example, under Mendelian expectations, an A female mated to an AB male should produce male offspring that carry either the AA or AB karyotype at a 50 : 50 ratio, and female offspring that carry the A or B haplotype at a 50 : 50 ratio (*n* offspring: 683).

### (e) Statistical analysis

All statistical analyses were conducted in R (v. 4.2.1) [41]. We adopted a Bayesian framework using the ‘brms’ package [42] for the first four reproductive measures described above: (i) egg production; (ii) egg fertility and early embryo development; (iii) hatching success of developing eggs; and (iv) offspring sex ratio.

For each reproductive measure, we fitted two separate multilevel models. The first model functioned to test the effects of female haplotype and parental haplotype combination on reproduction. This model included female haplotype as a fixed effect, and a second fixed effect variable termed ‘haplotypes shared’, which refers to the number of inversion haplotypes shared between the male and female, and therefore the degree of genetic similarity at the inversion. For example, a female with haplotype A shares two haplotypes with an AA male, one with AB/AC males, and none with BB/BC/CC males. Attempting to include a direct interaction between male karyotype and female haplotype would be inappropriate owing to the sheer number of possible contrasts and our limited *a priori* knowledge regarding how specific combinations would perform. However, if parental haplotype combination affects reproductive success, we would expect this effect to vary based on the number of haplotypes shared between parents. This model also allows us to interpret the main effect of female haplotype on reproduction, and whether any effect of parental haplotype sharing varies by female haplotype.

The model described above may miss some variation explained by specific male karyotypes. To account for this, we performed a second model including male karyotype as a separate fixed effect (with female haplotype included as a control). For every model, male and female age (scaled and mean centred) were also included as fixed effects, and male and female identity (ID) included as hierarchical groups. Whilst housing conditions were kept relatively consistent across the lifetime of the study population, the large timescales over which this population was kept meant that certain aspects of husbandry varied over time (such as food, conspecific identity and keeper identity). To ensure any effects of husbandry/environmental conditions were controlled for, we included a categorical variable of female birth year as an additional hierarchical group (17 levels, spanning 1997–2013).

Choice of family for Bayesian models was based on a combination of prior knowledge of the data and assessment of model fit using posterior predictive checks. For the ‘egg fertility and early embryo development’, ‘hatching success of developed eggs', and ‘sex ratio’ models, a binomial family was chosen. For the ‘egg production’ analysis, all variations of a Poisson distribution, negative binomial and negative binomial hurdle models fit the data very poorly with an extremely poor recovery of the tail ends of the distribution. A continuation ratio family (cRatio) (a highly flexible ordinal model) can be considered appropriate when used with data that is bounded and sequential (the attainment of one level is required for the attainment of the next). cRatio models have been proposed and used in the past for similar ordinal-like count data [43–45], and make logical sense in our case (see the electronic supplementary material, S5 for details). The use of a cRatio model also allows us to tease apart the separate probabilities associated with the attainment of each level, providing us with superior inference over a Poisson or negative binomial model. We present here the results of the cRatio model (see the electronic supplementary material, S5A-C for a summary of additional modelling attempts using more traditional approaches).

Regularizing zero-centred diffuse priors were chosen for all parameters. Models were run across four chains with 2000 warmup and 1000 post warmup iterations, except for the ‘hatching success of developed eggs' models which required 5000 warmup and 2500 post warmup iterations to reach a sufficient effective sample size. Model convergence was assessed visually using trace plots and parameter Ȓ values, none of which exceeded 1.01 indicating good convergence of between- and within-chain estimates. Posterior predictive checks were used to assess the adequacy of model fit.

We used the probability of direction (PD) to determine effect presence and direction, and the 90% highest density interval (HDI) to determine the degree of effect uncertainty. We chose a 90% rather than 95% HDI, because 90% has been suggested to be more stable [46]. The PD describes the proportion of the posterior distribution that is of the median's sign (i.e. a PD of 100% indicates all iterations were either positive or negative). The PD therefore provides the probability that a parameter is either positive or negative. As per convention, we consider PD values greater than 97.5% as substantial evidence of an effect (in combination with an assessment of the 90% HDI), because a PD of 97.5 is highly correlated with a two-sided *p-*value of 0.05. Effect direction is then evident by the sign of the median. All model checks and PD plots can be found in the electronic supplementary material. We performed a variance ratio analysis for each model, to calculate the variance explained by the hierarchical structure of the models (i.e. the ‘random effects’ – mother ID, father ID and mother birth year).

For the final reproductive measure, ‘offspring genotype’, we performed an exact binomial test for each combination of parental genotypes, to determine whether offspring genotype differed significantly from Mendelian expectations. Significance from binomial tests was determined by *p-*values of less than 0.05.

## 3. Results

### (a) Egg production

**Figure 2.**
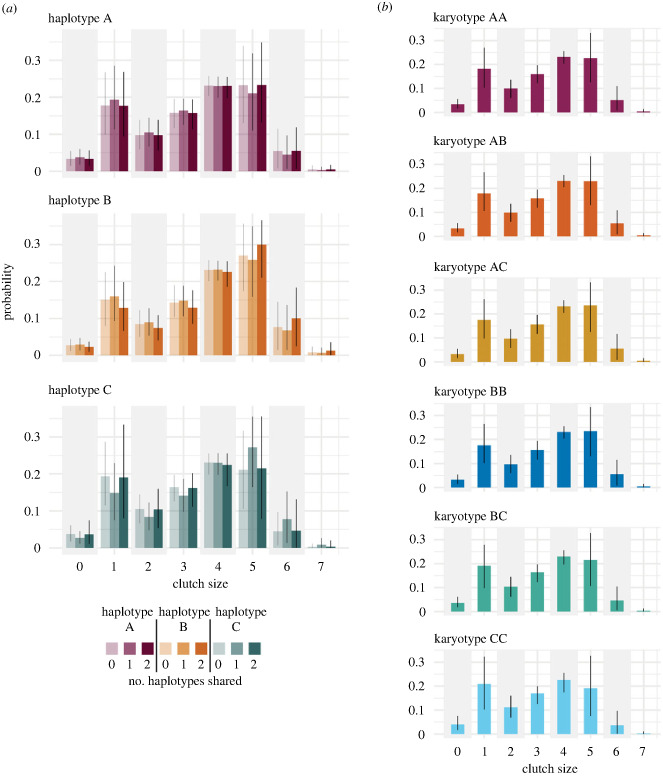
(*a*) The probability of laying different clutch sizes with respect to female inversion haplotype: A (purple); B (orange) and C (green), and the degree of parental haplotype sharing: no haplotypes shared (lightest tone), one haplotype shared (medium tone) and two haplotypes shared (darkest tone). Separate probabilities are given for each step-increase in clutch size. (*b*) Variation in the probability of laying different clutch sizes with respect to male inversion karyotype. Panels are split by male karyotype: AA (purple); AB (orange); AC (yellow); BB (dark blue); BC (green) and CC (light blue). Bars represent the posterior medians and error bars are the 90% HDI for the average individual. For a boxplot of the raw data see the electronic supplementary material, figure S1*d.*

We found no evidence of an effect of either female inversion haplotype, haplotype sharing (the number of haplotypes shared between the male and female, i.e. their genetic similarity at the inversion) or male karyotype on egg production (figure 2; electronic supplementary material, figure S1*a*–*h*). The effect of haplotype sharing was not influenced by female haplotype (figure 2*a*; electronic supplementary material, figure S1*c*).

Across all haplotypes, the most probable clutch size was four or five eggs, after which a single-egged clutch was more probable than any other clutch size. Very few clutches produced more than seven eggs (figure 2*a*). The high number of single-egged clutches is probably the result of an age-related decline in clutch size: we found a strong negative effect of female (but not male) age on egg production (est. = −0.67; 90% HDI: −0.79, −0.52; PD = 100%; electronic supplementary material, figure S1*e*,*f*). As females aged, there was an increase in the probability of laying a single-egged clutch, and a strong (but smaller) increase in the probability of laying no eggs, while the probability of each step-increase in clutch size also declined with age (none of these age effects were dependant on female inversion haplotype). A summary of the variance decomposition analysis is presented in the electronic supplementary material, table S1.

### (b) Egg fertility and early embryo development

**Figure 3.**
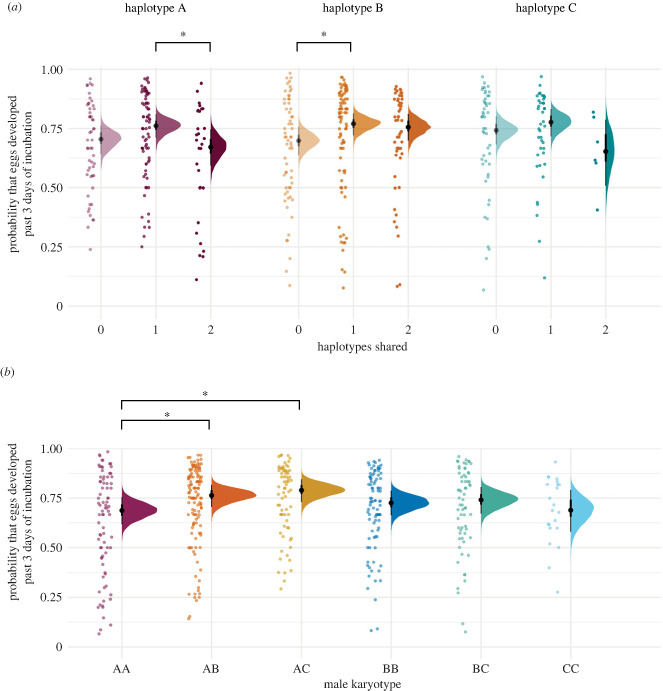
(*a*) The probability that eggs will survive early development (relative to being either infertile or dying during early development), with respect to female inversion haplotype: A (purple), B (orange) and C (green), and the degree of parental haplotype sharing: no haplotypes shared (lightest tone), one haplotype shared (medium tone) and two haplotypes shared (darkest tone). Asterisks indicate that the model observed a substantial difference between connections (PD > 97.5%) (A females sharing two haplotypes relative to one haplotype, B females sharing one haplotype relative to no haplotypes). (*b*) The probability that eggs will be fertilized and survive early development, with respect to male inversion karyotype: AA (maroon), AB (orange), AC (yellow), BB (dark blue), BC (green) and CC (light blue). Asterisks indicate that the model observed a substantial difference between connections (PD > 97.5%) (AB and AC relative to AA male karyotypes). Points show the raw observed data, density plots show the posterior distributions (and median and 90% HDI) for the average individual. Points have been jittered to aid visualization and prevent overlaying points.

We found no evidence of an effect of female haplotype on the probability of an egg developing past day 3 of incubation (figure 3*a*; electronic supplementary material, figure S2*a*,*b*). Overall, we found no consistent effect of parental haplotype sharing on early embryo development. There were, however, two exceptions: firstly, a clear but small positive effect for B females that shared one haplotype with the male, relative to B females that shared no haplotypes with the male (median = 0.37; 90% HDI: 0.06, 0.66; PD = 97.9%). Specifically, B : AB and B : BC parental combinations produced eggs that were around 40% more likely to be fertilized and survive early development relative to B : AA, B : CC or B : AC parental combinations (median_(odds ratio)_ = 1.44). Secondly, we found a clear negative effect for A females that shared two haplotypes with the male, relative to A females that shared one haplotype with the male (median = −0.45; 90% HDI: −0.81, −0.08; PD = 97.8%). Specifically, A : AA parental combinations were around half as likely to produce eggs that were fertilized and survived early development relative to A : AB and A:AC parental combinations (median_(odds ratio)_ = 0.63) (figure 3*a;* electronic supplementary material, figure S2*c*).

We found no consistent effect of male karyotype on egg fertility and early embryo development. However, we did find a small but clear negative effect of AA males relative to AB males (median = −0.38; 90% HDI: −0.66, −0.07; PD = 97.8%) and AC males (median = −0.54; 90% HDI: −0.86, −0.17; PD = 99.6%) (figure 3*b;* electronic supplementary material, figure S2*f*,*g*). Specifically, eggs from females paired to AA males were around half as likely to be fertilized or survive early development, relative to eggs from females paired with AB and AC males (median_(odds ratio)_ = 0.68 and 0.58 respectively). AB and AC carrying males did not differ substantially from any other male karyotype. There was no clear effect of male or female age on egg fertility and early embryo development (electronic supplementary material, figure S2*d*–*e*). A summary of the variance decomposition analysis is presented in the electronic supplementary material, table S1.

### (c) Hatching success of developing eggs

**Figure 4.**
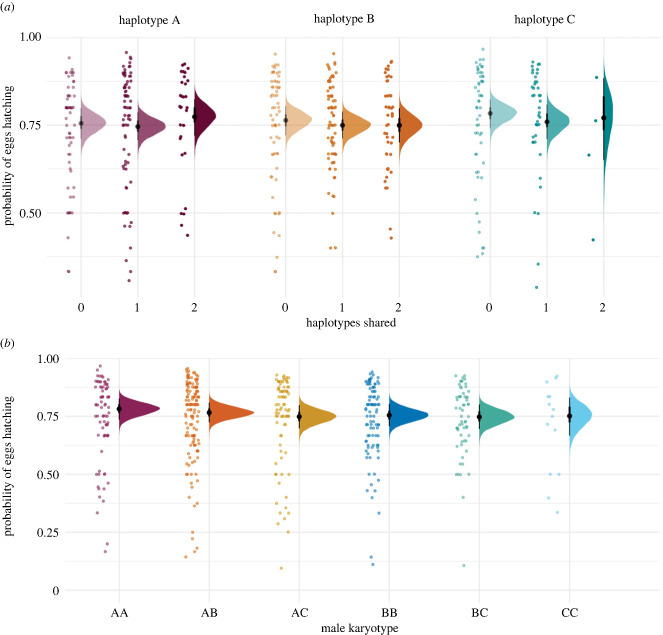
(*a*) Variation in the probability that a developing egg (containing an embryo at day 3 of incubation) will survive to hatch, with respect to female inversion haplotype: A (purple), B (orange) and C (green), and the degree of parental haplotype sharing: no haplotypes shared (lightest tone), one haplotype shared (medium tone) and two haplotypes shared (darkest tone). (*b*) Variation in the probability that a developing egg will survive to hatch, with respect to male inversion karyotype: AA (maroon), AB (orange), AC (yellow), BB (dark blue), BC (green) and CC (light blue). Points show the raw observed data, density plots show the posterior distribution (and median and 90% HDI) for the average individual. Points have been jittered to aid visualization and prevent overlaying points.

We found no evidence of an effect of female haplotype, haplotype sharing or male karyotype on the hatching success of developing eggs (i.e. those that showed evidence of development at day 3 of incubation) (figure 4; electronic supplementary material, figure S3*a*–*g*). The effect of haplotype sharing was also not influenced by female haplotype (figure 4*a*; electronic supplementary material, figure S3*c*). We also found no evidence of an effect of male or female age on hatching success (electronic supplementary material, figure S3*d,e*). A summary of the variance decomposition analysis is presented in the electronic supplementary material, table S1.

### (d) Offspring sex ratio

**Figure 5.**
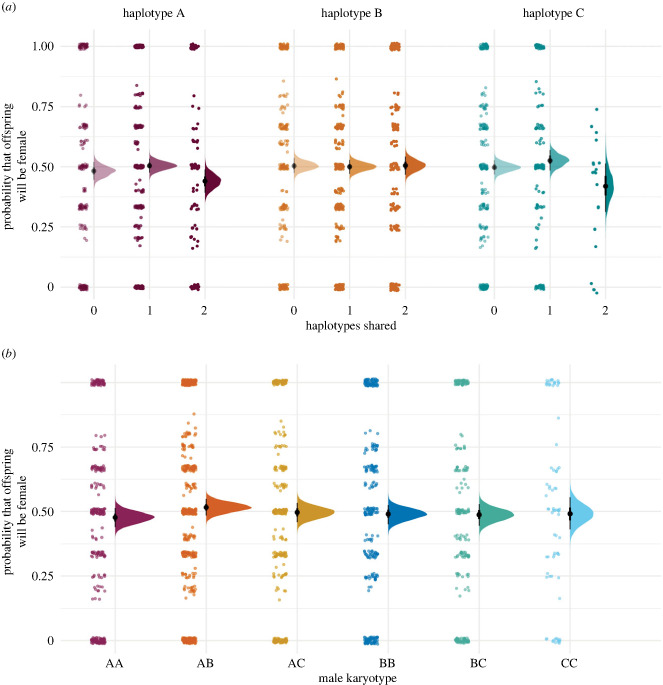
(*a*) Variation in the probability that offspring will be female (relative to male) with respect to female inversion haplotype: A (purple), B (orange) and C (green), and parental haplotype sharing: no haplotypes shared (lightest tone), one haplotype shared (medium tone) and two haplotypes shared (darkest tone). (*b*) Variation in the probability that offspring will be female (relative to male), with respect to male inversion karyotype: AA (maroon), AB (orange), AC (yellow), BB (dark blue), BC (green) and CC (light blue). Points show the raw observed data, density plots show the posterior distribution (and median and 90% HDI) for the average individual. Points have been jittered to aid visualization and prevent overlaying points.

We found no evidence of an effect of female haplotype, the degree of haplotype sharing or male karyotype on the sex ratio of offspring (figure 5; electronic supplementary material, figure S4*a*–*g*). The effect of haplotype sharing was not influenced by female haplotype (figure 5*a*; electronic supplementary material, figure S4*c*), and there was also no evidence of an effect of male or female age (electronic supplementary material, figure S4*d,e*)). A summary of the variance decomposition analysis is presented in the electronic supplementary material, table S1.

### (e) Offspring genotype ratios

There was no evidence of an effect of parent genotypes on expected offspring genotype ratios, with Mendelian expectations being met for both female and male offspring, and for all combinations of parent genotype (table 1).

**Table 1.** Expected and observed offspring genotype ratios for each combination of parental genotypes. (Also provided are the *p*-values and 95% confidence intervals (CI) from binomial exact tests assuming the Mendelian expectation of a 50 : 50 ratio within each combination.)

parent genotype combination (female : male)	offspring sex	expected offspring genotype	number of offspring	observed proportion of offspring genotypes	exact binomial test for Mendelian expectations	
					*p*	95% CI
A : AB	male	AA, AB	29, 21	0.58, 0.42	*p* = 0.32	0.43–0.72
	female	A, B	42, 42	0.5, 0.5	*p* = 1	0.39–0.61
A : AC	male	AA, AC	21, 20	0.51, 0.49	*p* = 1	0.35–0.67
	female	A, C	15, 28	0.35, 0.65	*p* = 0.07	0.21–0.51
A : BC	male	AB, AC	13, 8	0.62, 0.38	*p* = 0.38	0.38–0.82
	female	B, C	26, 23	0.41, 0.59	*p* = 0.34	0.26–0.58
B : AB	male	AB, BB	29, 31	0.48, 0.52	*p* = 0.90	0.35–0.62
	female	A, B	28, 38	0.42, 0.58	*p* = 0.27	0.30–0.55
B : AC	male	AB, BC	16, 7	0.7, 0.3	*p* = 0.10	0.47–0.87
	female	A, C	14, 14	0.5, 0.5	*p* = 1	0.31–0.69
B : BC	male	BB, BC	24, 13	0.65, 0.35	*p* = 0.10	0.47–0.80
	female	B, C	18, 14	0.56, 0.44	*p* = 0.60	0.38–0.74
C : AB	male	AC, BC	14, 11	0.56, 0.44	*p* = 0.69	0.35–0.76
	female	A, B	22, 28	0.44, 0.56	*p* = 0.48	0.30–0.59
C : AC	male	AC, CC	6, 12	0.33, 0.67	*p* = 0.24	0.13–0.59
	female	A, C	18, 24	0.43, 0.57	*p* = 0.44	0.28–0.59
C : BC	male	BC, CC	9, 5	0.64, 0.36	*p* = 0.42	0.35–0.87
	female	B, C	5, 5	0.5, 0.5	*p* = 1	0.19–0.81

## 4. Discussion

Despite the strong effects of the Z chromosome inversion polymorphism for male fertility in zebra finches, we found no evidence that inversion haplotypes contain alleles with differential effects on egg production by females or survival of embryos through to hatching. Our results indicate it is unlikely that sexually antagonistic alleles acting upon egg production (a female-specific reproductive trait) have accumulated within the inversion. Instead, our findings suggest either: (i) the Z chromosome inversion haplotypes explain little variation in egg production, because any between-haplotype variation results in very similar mean phenotypes; (ii) prior sexual antagonism associated with egg production has already been resolved (for example through sex-biased gene expression [47]); (iii) there are relatively few genes responsible for variation in egg production located in the inversion, but instead they are located elsewhere on the Z or on the autosomes; (iv) egg production is a highly polygenic trait, and any associated loci located in the inversion exhibit a small proportion of genetic variation relative to loci elsewhere in the genome; or egg production may simply exhibit very little genetic variation.

Given the accumulated differences between inversion haplotypes, we analysed whether parental genotype combination is likely to contribute to variation in reproductive traits. We found no evidence that inversion haplotype sharing has a consistent effect on reproductive traits. We did, however, find that the probability of offspring surviving early development (relative to an egg being unfertilized or an embryo dying before 3 days of development), varied depending on the number of haplotypes shared and female haplotype. Specifically, ‘B’ females sharing one haplotype with the male (B : AB and B : BC combinations) had a higher likelihood of producing eggs that developed beyond 3 days of incubation, relative to ‘B’ females sharing no haplotypes with the male (B : AA, B : CC and B : AC combinations). Additionally, ‘A’ females sharing two haplotypes with the male (A : AA combinations) had a higher chance of eggs failing to develop, relative to ‘A’ females sharing one haplotype with the male (A : AB and A : AC combinations) (figure 3*a*). However, if parental haplotype sharing was consistently important, we would accordingly expect to observe a consistent effect associated with each degree of haplotypes shared. Our results do suggest that some specific combinations of parental genotypes perform better than others, but we suspect this is predominantly driven by the effect of male karyotype, because we also found that relative to homokaryotypic (AA carrying) males, heterokaryotypic (AB and AC carrying) males are more likely to produce fertilized eggs that survive beyond 3 days of development (figure 3*b*). This is additional to the prior evidence that AB and AC males have the fastest and most successful sperm under sperm competition [8,9]. Since multiple mating was not permitted in our population, the improved egg survival we observed must be independent of sperm competition, instead suggesting an additional non-competitive fertilization and/or developmental advantage. Our data do not allow us to discriminate between fertilization failure and embryo death as the cause of early reproductive failure in undeveloped eggs, but our findings do suggest that variation in male fertilization success may underpin the effects of haplotype sharing on egg development that we observed here. While female inversion haplotype does not appear to affect our measured reproductive traits, whether it influences other aspects of fitness remains unknown.

In their original study, despite the strong effects of the male karyotype for sperm traits, Kim *et al*. [8] found no systematic evidence of an effect of male inversion karyotype on hatching success, although they were unable to account for the influence of CC males. Conversely, Knief *et al.* [6] presented tentative evidence that mortality rates may be slightly higher in the offspring of heterokaryotypic males. Neither study accounted for the influence of female haplotype, or the interaction between male and female genotypes. Additionally, in both studies, measures of unhatched eggs represented those that failed at any stage from fertilization to late development. Our study—which does consider the influence of female haplotype, their interaction with male karyotype, and CC males, as well as partitioning hatching success into two distinct functional categories (representing separate and mechanistically divergent stages of hatching failure)—finds that aside from the positive effect of AC and AB males relative to AA males, there is no further evidence that male karyotype has a systematic influence over any other measures of reproductive success. Our finding that AB and AC males may actually carry an advantage during fertilization and/or early embryo development, lends additional support to the evidence put forward by both Kim *et al.* [8] and Knief *et al.* [9] that variation between haplotypes is maintained in the population by a heterozygote advantage for male fitness.

In some systems, females employ post-copulatory reproductive strategies to offset the costs associated with mating with less fit males [16,20,21] (e.g. those homozygous for the inversion, who have more poorly performing sperm). We explored this here by analysing whether offspring sex and genotype ratios varied by parental haplotype sharing. For example, we might expect females to favour producing a female-biased brood when mated to homozygous males that share her haplotype (i.e. in which case all her male offspring would be less fit). However, we found no evidence that offspring sex ratio was influenced by inversion haplotype, or the number of haplotypes shared between parents. Similarly, offspring genotype ratios conformed to Mendelian expectations under all parental combinations. It is therefore unlikely in this system that females employ post-reproductive differential offspring investment based on the father's inversion haplotype. Differential investment in offspring sex ratio is predicted to occur where the costs and benefits of producing offspring differ between sons and daughters [16,48,49]. In the zebra finch, females have previously been shown to employ facultative offspring sex ratio adjustments based on female condition [22], and differential offspring investment has also been observed based on partner quality [20]. It is possible that we do not observe differential investment here because conflict over optimal trait values limits the ability of females to drive such traits to fixation. Alternatively, because male karyotype has no systematic influence on female reproductive traits or on offspring survival, instead primarily influencing sperm success under sperm competition, the strength of selection for such a trait will also probably depend on the degree of extra pair paternity. Finally, if inversion haplotype is linked with genes for phenotypic or behavioural signals associated with attractiveness in males (for example male band colour [50]) females may select the more suitable males based on pre-copulation cues, providing little need to evolve a mechanism for adjusting investment post-mating. Beak length has already been found to be linked with male Z inversion karyotype in this species, providing one potential mechanism for pre-copulatory female choice [6]. However, since natural mate choice was not permitted in our study population, we were unable to test this.

Finally, although not related to the inversion polymorphism, we observed a clear decline in egg production with female age, which is consistent with general trends of reproductive senescence across many (though not all) female birds [51]. We also observed a marked increase in the number of single-egged clutches with female age (electronic supplementary material, figure S1*f*). It may be that for older females, reproductive fitness is maximized by concentrating effort into the formation and post-laying care of a single egg, rather than spreading limited energy over a larger clutch. Laying larger clutches may be physically very challenging for older birds that have probably experienced a reduction in their follicular reserve, a decline in normal immunological and hormonal functioning, and an increase in the energy required for somatic maintenance [52]. This effect became apparent in our population when female birds reached between 3 and 4 years of age. In the wild, mortality is high, and the median life expectancy is only approximately four months [53]. It therefore seems unlikely that this phenomenon would be observed in the wild, but whether it is a specific feature of our population, or a more general pattern observed across captive birds is also unknown.

Understanding the factors that contribute to variation in reproductive success is a key goal of evolutionary research. Our findings provide a thorough test of the effects of a sex-linked inversion polymorphism for reproductive traits. We have shown that this inversion polymorphism, or ‘supergene’, despite its important effects for male fertility, does not appear to influence egg production by females or survival of embryos through to hatching. We have also provided additional evidence that the zebra finch Z inversion is probably being maintained in a stable polymorphism owing to heterozygote advantage for male fitness, whereby heterokaryotypic males carrying one copy of the ancestral inversion not only have faster and more successful sperm under sperm competition, but may also have an increased likelihood of fertilization and/or early embryo survival relative to homokaryotypes.

## Data Availability

Data and code are available in the Dryad Digital Repository: https://doi.org/10.5061/dryad.cz8w9gj7w [38]. Electronic supplementary material is provided online [54].
